# Left Ventricular Global Function Index and the Impact of its Companion Metric

**DOI:** 10.3389/fcvm.2021.695883

**Published:** 2021-08-30

**Authors:** Rienzi A. Diaz-Navarro, Peter L. M. Kerkhof

**Affiliations:** ^1^Department of Internal Medicine and Center for Biomedical Research, School of Medicine, Universidad de Valparaiso, Valparaiso, Chile; ^2^Department of Radiology & Nuclear Medicine, Amsterdam University Medical Centers, Amsterdam, Netherlands

**Keywords:** global function index, ratio-based metric, ejection fraction, stroke volume, end-systolic volume, left ventricular mass, ratiology, ventriculo-arterial coupling

## Abstract

Left ventricular (LV) global function index (LVGFI) has been introduced as a volume-based composite metric for evaluation of ventricular function. The definition formula combines stroke volume (SV), end-systolic volume (ESV), end-diastolic volume (EDV) and LV mass/density. Being a dimensionless ratio, this new metric has serious limitations which require evaluation at a mathematical and clinical level. Using CMRI in 96 patients we studied LV volumes, various derived metrics and global longitudinal strain (GLS) in order to further characterize LVGFI in three diagnostic groups: acute myocarditis, takotsubo cardiomyopathy and acute myocardial infarction. We also considered the LVGFI companion (C), derived from the quadratic mean. Additional metrics such as ejection fraction (EF), myocardial contraction fraction (MCF) and ventriculo-arterial coupling (VAC), along with their companions (MCFC and VACC) were calculated. All companion metrics (EFC, LVGFIC, MCFC, and VACC) showed sex-specific differences, not clearly reflected by the corresponding ratio-based metrics. LVGFI is mathematically coupled to both EF (with R = 0.86) and VAC (R = 0.87), which observation clarifies why these metrics not only share similar prognostic values but also identical shortcomings. We found that the newly introduced LVGFIC has incremental value compared to the single use of LVGFI, EF, or GLS, when characterizing the three patient groups.

## Introduction

The left ventricular (LV) global function index (GFI) has been introduced by Mewton et al. as a novel metric that integrates LV structure with global function, and was shown to be a powerful predictor of incident heart failure and hard cardiovascular events (1). It is stated that a higher LVGFI reflects better LV cardiac performance (2). Essentially, LVGFI combines stroke volume (SV), end-systolic volume (ESV), and end-diastolic volume (EDV) in the following formula:

(1) $$\begin{array}{l} \text{LVGFI}=\text{SV }/\;\left[\text{LVmass}/\text{q }+\;\left(\text{ESV }+\text{ EDV}\right)/2\right] \end{array}$$

where LVmass is normalized to myocardial density (q), so that the term (LVmass/q) carries the dimension of volume. As a result, the expression for LVGFI refers to a metric without any physical dimension. The ratio is not unique, as several LV volume combinations yield the same outcome, e.g., the pair with ESV = 50 and EDV = 100, as well as the combination ESV = 80 and EDV = 140 mL, lead to an LVGFI value of 28.6 % when LVmass/q is kept at 100. This fact implies that LVGFI, in order to be meaningful, requires consideration of a companion (C), denoted as LVGFIC (3). In this study we employ the quadratic mean as a suitable candidate for C (3), as will be explained later.

A further problem with LVGFI resides in the fact that within the denominator the contribution by LVmass/q cannot be distinguished from the (ESV+EDV)/2 component, i.e., if LVmass/q increases, then a balanced decrease of (ESV+EDV)/2, realized while keeping SV unaltered, makes that the denominator and therefore LVGFI remain unchanged. For example:

State 1: LVmass/q = 100, ESV = 50, EDV = 100, (ESV+EDV)/2 = 75, denominator = 175 mL
State 2: LVmass/q = 110, ESV = 40, EDV = 90, (ESV+EDV)/2 = 65, denominator = 175 mL,

while, of course, the two states are cardiomechanically different (4).

The limitation as mentioned for the sum of LVmass/q and (ESV+EDV)/2, does not apply to the constant value of the sum of ESV and EDV, as any change of ESV and EDV (while keeping their sum constant) would be visible in the numerator SV. Some of these difficulties are eliminated in the myocardial contraction fraction (MCF) definition (5), where the term defined as (ESV+EDV)/2 is not included.

An attractive feature of C is the fact that it carries a physical dimension, namely volume. It is now evident that the newly introduced C metric plays a central role in the evaluation of LV function. This study explores the impact of LVGFIC in three diagnostic groups of patients with acute cardiac problems. Additionally, we will analyze the mathematical aspects of the definition formula, as various terms included are not independent, e.g., SV = EDV–ESV, while the term (ESV+EDV) correlates with the traditional metric ejection fraction (EF).

## Methods

### Patients

This study included 96 patients consecutively admitted to the hospital because of suspected ST-segment elevation with myocardial infarction (STEMI). All patients presented with prolonged oppressive chest pain at rest (>30 min) with an ST-elevation >0.2 mV in two or more contiguous precordial leads and/or >0.1 mV in other leads, and elevated serum troponin level. All patients underwent invasive coronary angiography (ICA) performed using radial artery puncture. In patients with normal coronary arteries, CMRI was performed 72–96 h following the ICA, after ruling out acute kidney injury due to the use of iodinated contrast. Patients with previous myocardial infarction, other known heart disease or contraindications for CMRI were excluded from the study. The local Ethics Committee of Reñaca Clinic approved this study, and written consent was obtained from all patients.

### Cardiac Magnetic Resonance Imaging (CMRI) Studies

The CMRI studies were performed on a SIGNA Excite 1.5-Tesla resonator (General Electric Medical Systems, Milwaukee, MN, USA) with a multi-element phased-array coil. All patients were placed in a supine position, and images were acquired at an end-expiratory breath hold with ECG gating. Initial scout images were acquired to locate the heart. Studies involved two-dimensional fast imaging using steady-state acquisition sequences for cine-CMRI, which were used to assess segmental and global contraction of the heart according to the American Heart Association classification (6). To detect areas of high signal intensity compatible with myocardial edema, a short-tau inversion-recovery (STIR) imaging sequence was applied before administration of the contrast agent, gadolinium. To target areas of fibrosis, late gadolinium enhancement (LGE) CMRI sequences were obtained about 10 min after the administration of intravenous gadolinium (0.2 mmol/kg). The LGE images were acquired using an inversion-recovery gradient-recalled echo sequence. The inversion time was adjusted to null the signal from viable myocardium (7). In the analysis of LGE sequences, subendocardial, intramyocardial, subepicardial, and transmural delayed contrast enhancement (DCE) were considered.

The cine-CMRI, T2-STIR, and LGE sequences were obtained in the short-axis view covering the LV from the base to apex in the two-, three- and four-chamber views. The slice thickness was 8 mm with no slice gap, and the in-plane resolution was typically 1.5 mm × 1.5 mm. The temporal resolution of the cine-CMRI sequences was 20–30 frames per cardiac cycle.

Patients with subendocardial and transmural LGE were diagnosed with acute myocardial infarction (AMI). Patients who had subepicardial and/or intramyocardial DCE were diagnosed with myocarditis. Finally, Takotsubo cardiomyopathy (TCM) was diagnosed in patients exhibiting high T2-STIR sequences (myocardial edema) with absence of LGE in the late post-gadolinium sequences. CMRI studies were evaluated by two experts in this diagnostic technique. In case of disagreement, the differences were resolved by reaching consensus with a third expert observer.

A fully automated volumetric analysis of LV volumes, mass and function was performed using a novel deep learning-based algorithm within a dedicated commercially available software package (cvi42, Version 5.10.1, Circle Cardiovascular Imaging Inc.). Global myocardial LV strain (GLS) was measured from integrated long and short-axis and axis cine images, using feature tracking. All strain parameters were quantified offline by an experienced observer blinded to all patient data. LV endocardial and epicardial borders in short and long axis views were automatically determined. The right ventricular (RV) insertion points and mitral annular plane were specified. An automated tracking algorithm was applied and tracking of LV segments, as well as the mitral annular plane, was confirmed. Manual adjustments were performed as needed to optimize LV wall tracking. Basal, mid-ventricular, and apical short-axis images, as well as LV long-axis images were analyzed for global circumferential, radial and longitudinal strain measurements.

### Statistical Analysis

Data are presented as mean value, along with the corresponding standard deviation, and analyzed using IBM SPSS version 22 (IBM Corporation, Armonk NY). Comparison of means is based on t-statistics. The Fisher z-transform or the William's test is used to compare R-differences between groups, as appropriate. A one-sided *P* < 0.05 is considered statistically significant.

### Dissecting LVGFI and Defining Its Companion

In the definition formula LVGFI = SV / [LVmass/q + (ESV+EDV)/2] (see equation 1) we discern three components (apart from q): SV, LVmass and average LV volume. Note that the term (LVM/q) with q as myocardial density, has the physical dimension of volume, as q is expressed as 1.05 mL/g. As emphasized before, LVGFI is not unique and requires consideration of its companion metric, defined as the quadratic mean:

(2) $$\text{LVGFIC = }\sqrt{\;}\;{\left[{\left(\text{SV}\right)}^{\text{2}}\text{ +}\{\text{LVmass/q + }(\text{ESV+EDV})\text{/2})\right\}}^{\text{2}}]$$

More in general, the companion of any data pair x and y is calculated as the quadratic mean of x and y, where in our case for convenience the constant √(1/2) = 0.7071 has been omitted. This procedure yields the size of the hypotenuse for the perpendicular line segments x and y, as given by the familiar Pythagorean theorem (3). Obviously, x and y must have the same physical dimension(s). Thus, for the ratio Z = (y/x) the associated companion, denoted as ZC, is defined as:

(3) $$\begin{array}{l} \text{ZC}=\;\sqrt{\;}\left\{{\text{x}}^{2}\;+\;{\text{y}}^{2}\right\} \end{array}$$

**Figure 1:**
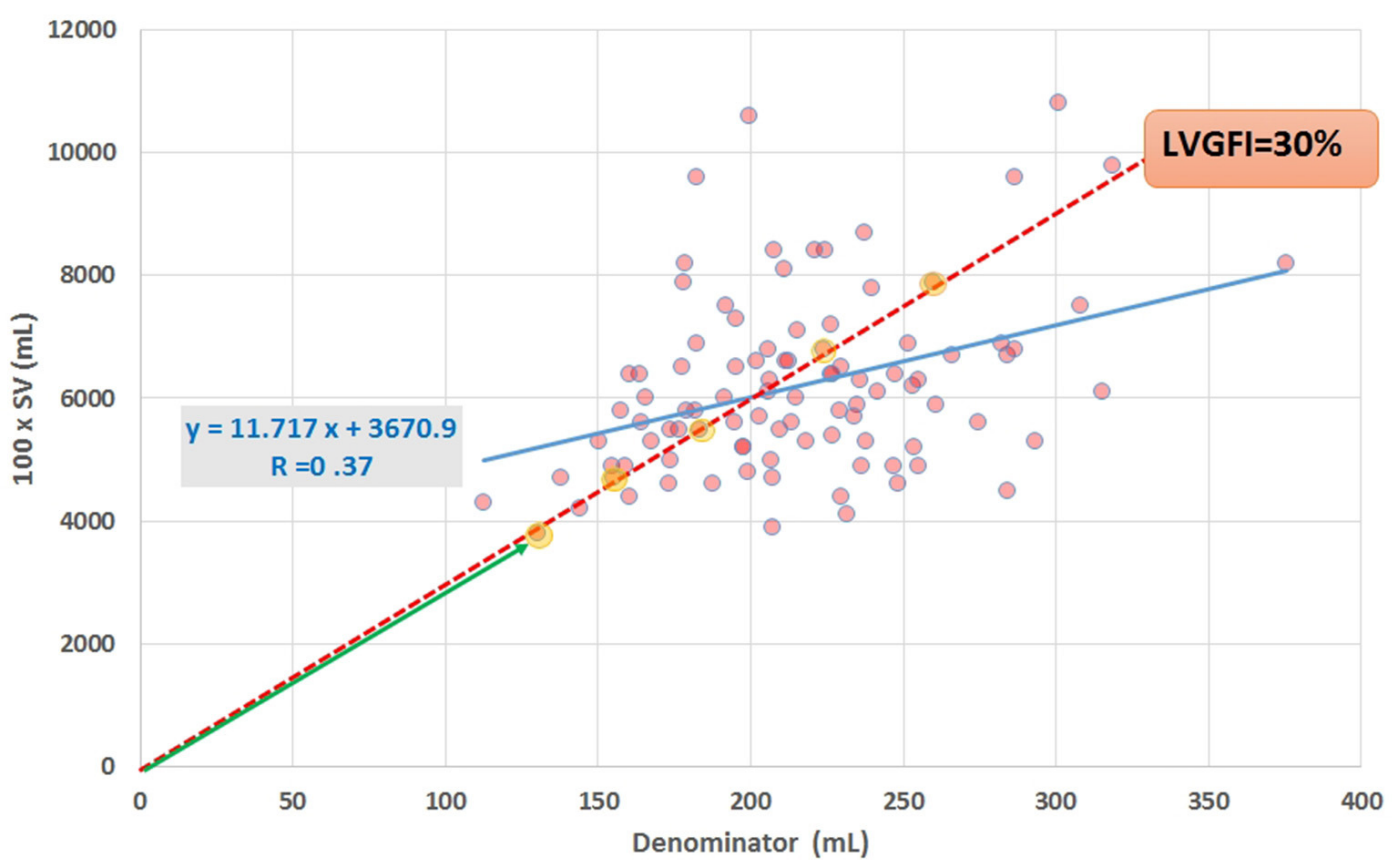
Scatter plot of data obtained by CMRI in 96 cardiac patients, showing SV (multiplied by 100 for scaling purposes) vs. the denominator in equation (1). Regression line (blue) and equation are included. The slope of the red broken line corresponds with the ratio formulated in equation (1), for the case that the ratio LVGFI is 30%. All (yellow marked) points on or very close to this line share the same value of the ratio, illustrating that the ratio itself is not unique. Further characterization of individual points requires specification of the corresponding hypotenuse (such as the length of the green line with arrow head for the lower-left point).

In this study we will apply the companion concept not only to LVGFI, but also to EF, ventriculo-arterial coupling (VAC) and MCF. Figure 1 shows the numerator (multiplied by 100 in order to express the ratio as a percentage) plotted against the denominator for all individuals under study, and illustrates that any single value selected for the slope LVGFI may refer to a wide variety of companion values. Also is demonstrated that the correlation between numerator and denominator is relatively low (R = 0.37 for *N* = 96), indicating that each component offers information that is to a large extent not generated by the other.

Essentially, the LVGFI refers to the ratio of SV and average overall LV size (i.e., the sum of averaged luminal and wall volume). On a beat-to-beat basis LVmass may be assumed to be fairly constant for a particular heart, implying that LVmass is a minor modulating factor in the denominator.

In our data set R = 0.55 for (ESV+EDV)/2 (range 49 to 196 mL) vs. LVmass (range 62 to 203 g), indicating that the two components in the denominator (equation 1) are far from entirely independent in this patient population (*N* = 96).

Apart from a comparison of LVGFI with EF, it is also of interest to compare LVGFI with the newly accepted strain-based metric GLS. Reportedly, treatment for 36 weeks with the sodium-glucose cotransporter 2 inhibitor empagliflozin diminished LV ESV (*P* = 0.021) and EDV (*P* = 0.005) in 42 patients with heart failure with reduced EF and type 2 diabetes or prediabetes, compared with a matched placebo group (*N* = 50), while LVmass, EF, GLS, and LVGFI did not change significantly (8). Thus, in selected cases the primary variables ESV and EDV may be of more importance than the derived metrics which are constructed upon a mathematical mixture (4).

## Results

**Figure 2:**
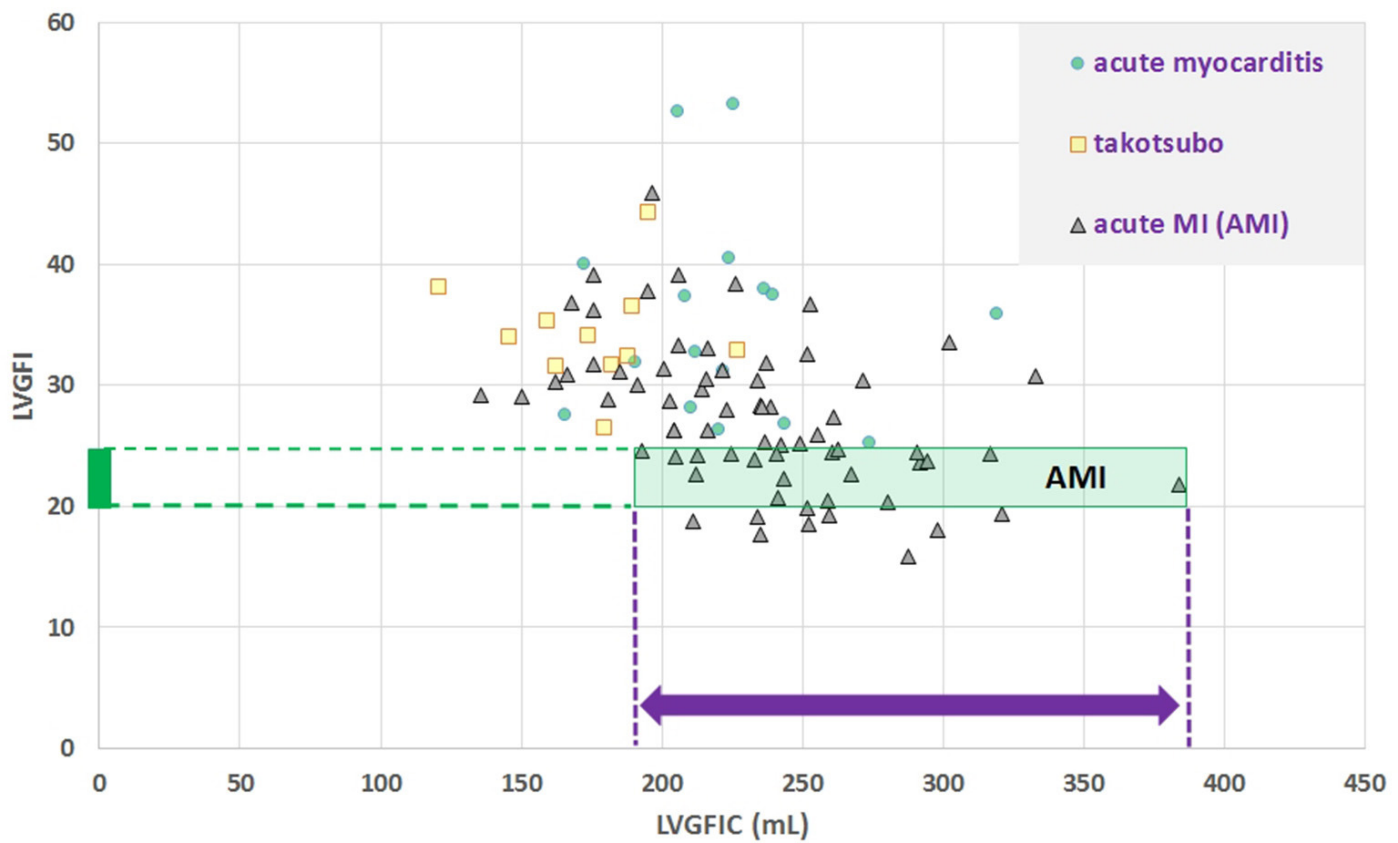
Scatter plot for dimensionless LVGFI vs. its companion metric LVGFIC (mL). The green rectangular area illustrates that e.g., for the narrow range 20<LVGFI<25 % this metric is not unique, as for the interval the individual data points are rather characterized by the prevailing LVGFIC (mL), with values between 193 and 384 mL. Correlations are not significant for the diagnostic subgroups, with the exception of acute myocardial infarction (MI), where R = −0.46 for *N* = 69.

**Figure 3:**
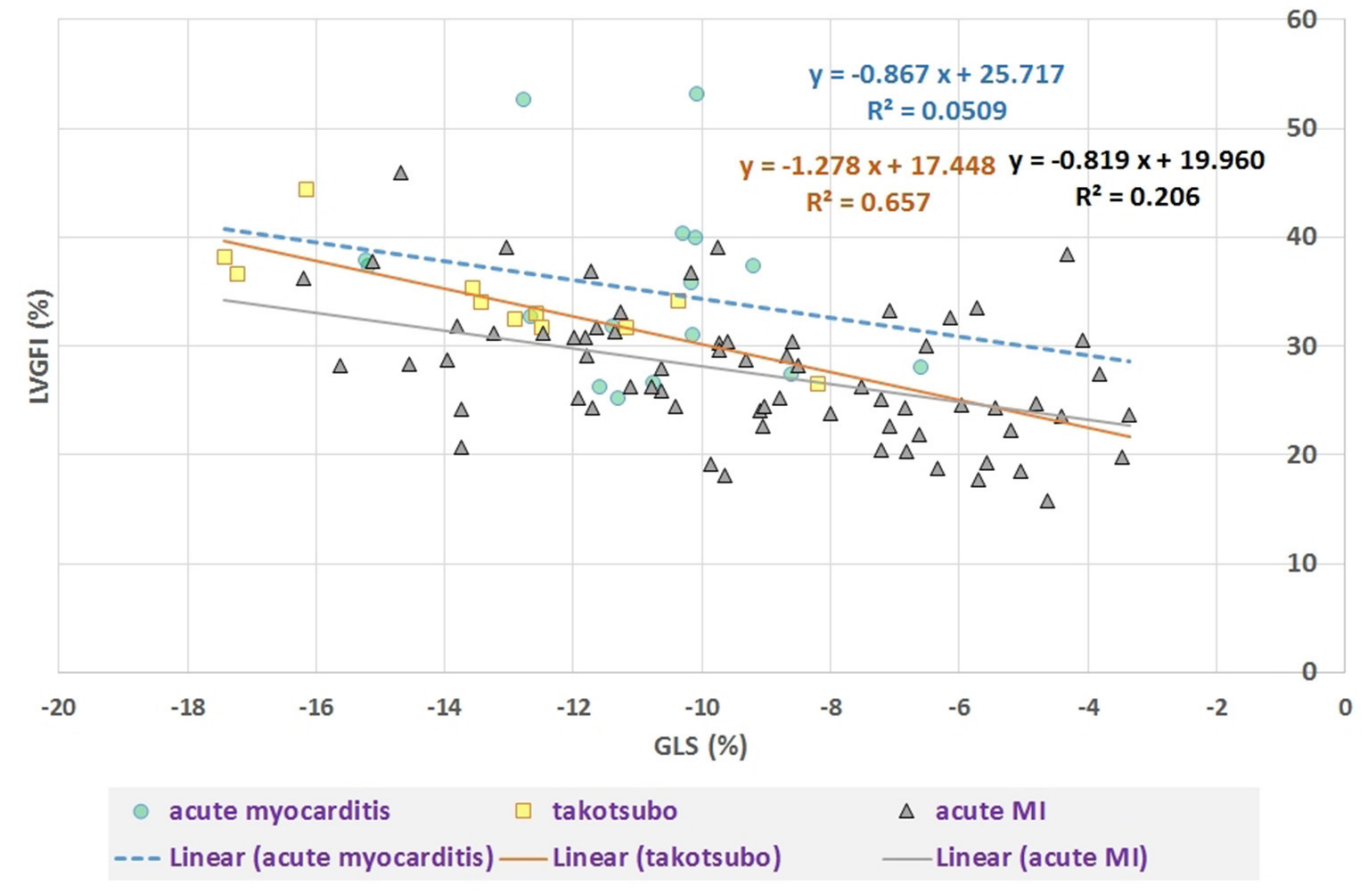
Scatter plot of LVGFI (%) vs. GLS (%). Overall correlation R = −0.51 (*P* < 0.0001), but after stratification for diagnostic group only a significant correlation (R = −0.81, *P* = 0.005) results for the takotsubo group (*N* = 11) and R = −0.45 (*P* < 0.001) for acute MI (*N* = 69). For acute myocarditis (*N* = 16) R = −0.23 (ns).

**Figure 4:**
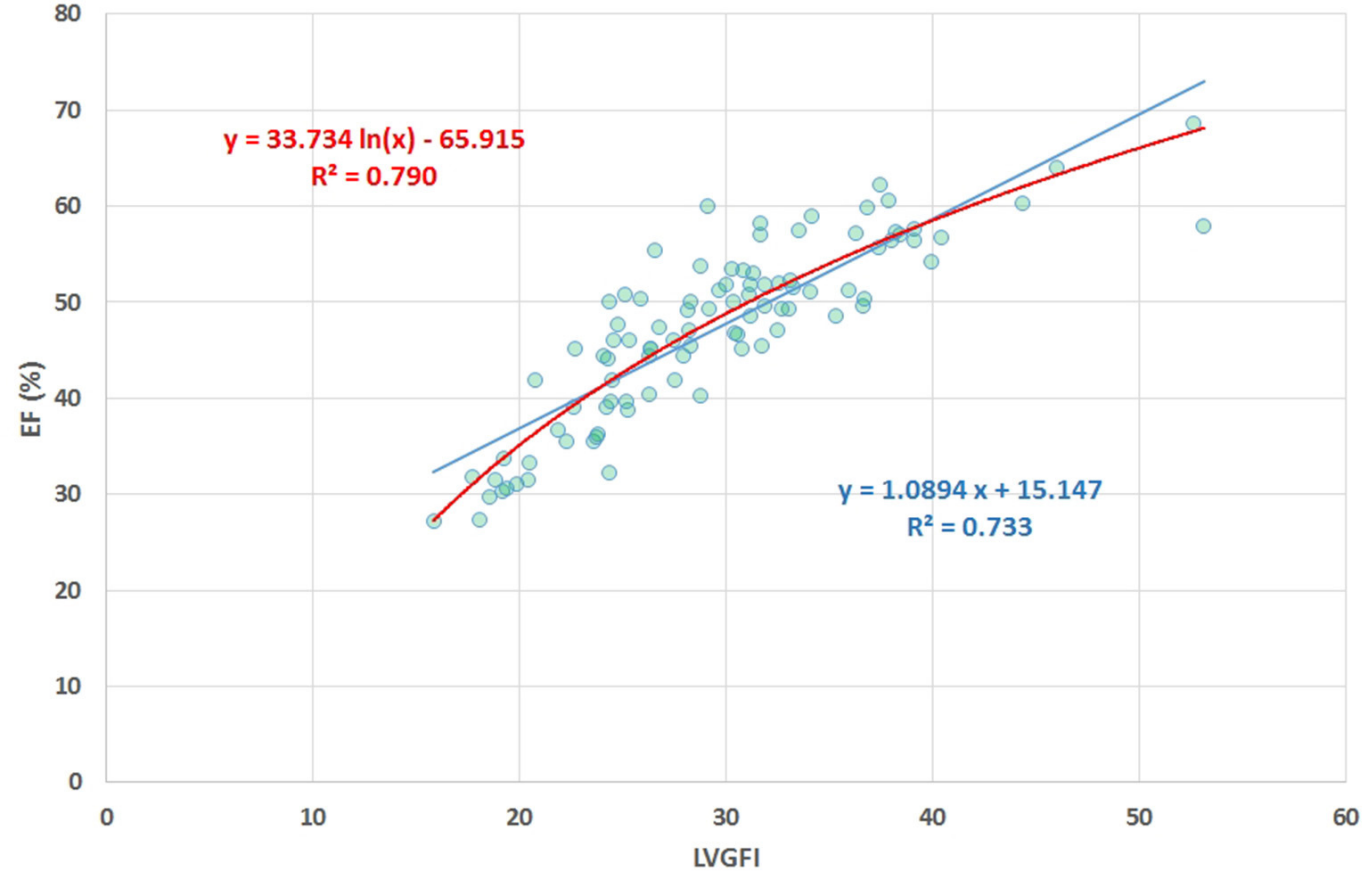
Scatter plot of EF (%) vs. LVGFI (%), yielding R = 0.86. The high correlation clearly demonstrates that the new metric LVGFI and the traditional EF are more or less equivalent, mainly due to the fact that they share the same components in their mathematical definitions.

**Figure 5:**
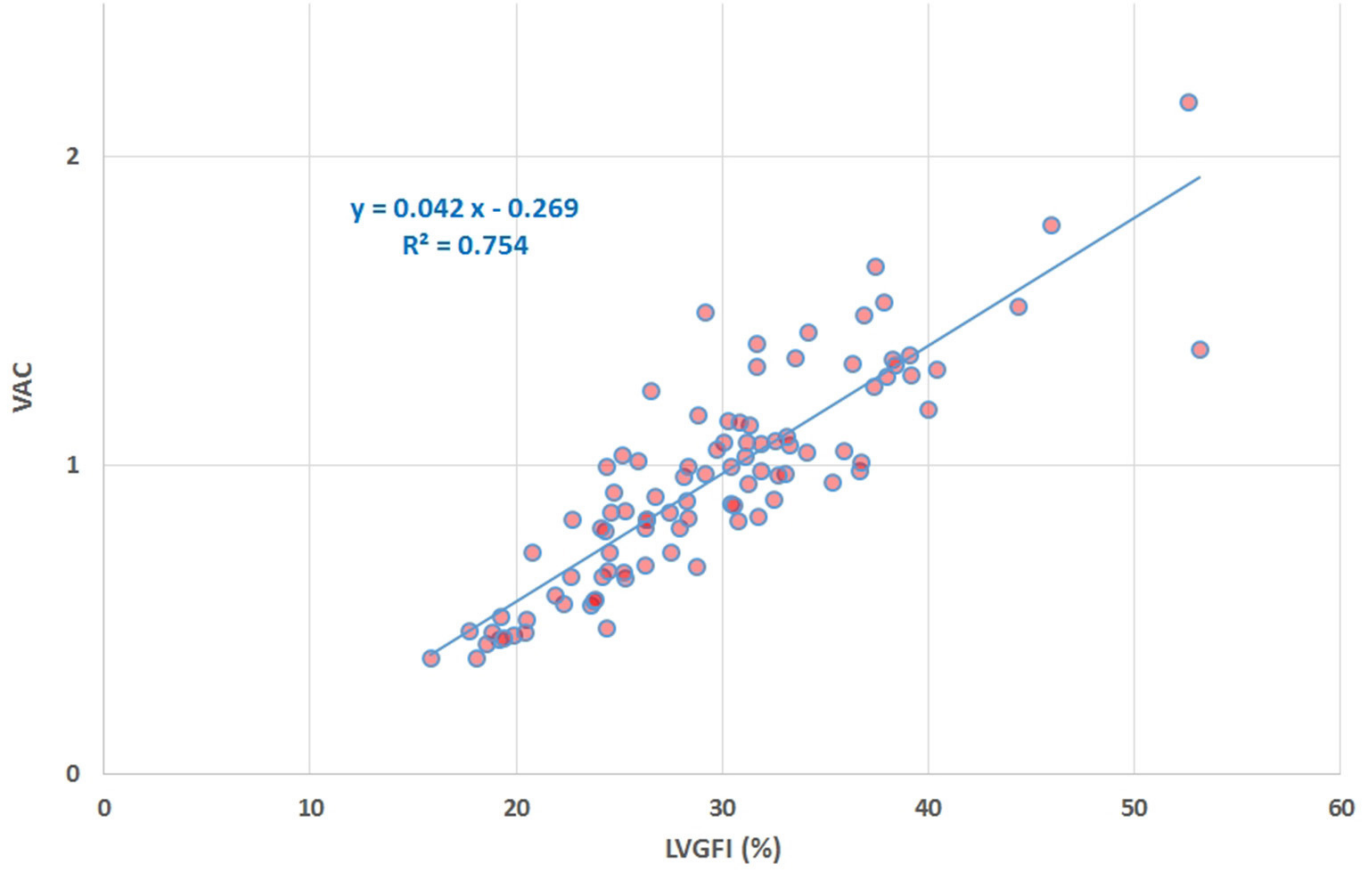
Scatter plot of VAC vs. LVGFI (%), yielding R = 0.87. The high correlation clearly demonstrates that the new metric LVGFI and the traditional VAC (assuming the intercept Vo of the systolic elastance vanishes) are more or less equivalent, mainly due to the fact that they share the same components in their mathematical definitions.

**Figure 6:**
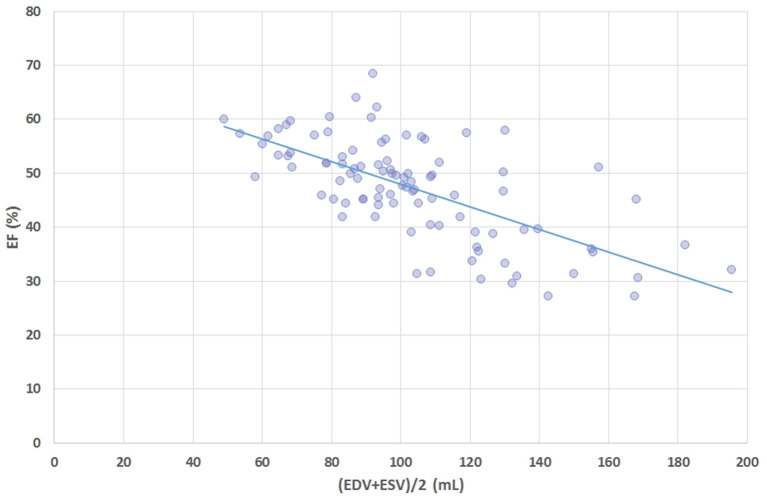
Scatter plot of ejection fraction (EF, %) vs. average volume i.e., (ESV+EDV)/2 shows that a major component of the definition formula for LVGFI is actually highly correlated (R = −0.66) with EF.

Sex-stratified patient characteristics including primary LV volume measurements and derived metrics are summarized in Table 1. Average values for LVmass, SV, ESV, and EDV are all significantly smaller in women compared to men. Ratio-based metrics such as EF, VAC, and LVGFI carry borderline significance when sexes are compared, in contrast to their associated companions (EFC, VACC, and LVGFIC). This means that the newly introduced complementary metrics detect differences that are not readily evident from the traditional ratio-based metrics. This enriching finding is also evident from a graphical presentation (Figure 2) showing that a relatively small range of LVGFI (e.g., between 20 and 25%) corresponds with a wide range of LVGFIC values that clearly reveal incremental details. The relatively low or even non-significant values found for the correlation coefficients referring to each diagnostic group emphasize that the associated companion metric carries information that is at least partly independent of the traditional ratio-based metric LVGFI. Similarly, for any specific value of LVGFI we find a wide range of GLS (Figure 3), again signifying that LVGFI does not fully capture fundamental information if indeed the importance of GLS is accepted. In contrast, the excellent correlation (R = 0.86 for the linear approach, which may be challenged by a non-linear fit such as the logarithmic example shown) with EF (Figure 4) suggests that these two metrics are almost equivalent, and invites a discussion about what LVGFI may add to what we can already learn from the simple and familiar EF. Likewise we found a high correlation (R = 0.87) with VAC (Figure 5), again questioning the incremental impact beyond existing widely used metrics. Furthermore, one essential component of LVGFI, namely (ESV+EDV)/2, is already significantly (R = −0.66) tied to EF (Figure 6), raising the question to what extend inclusion of SV and LVmass do materially enhance the discriminating power of LVGFI compared with EF. The companions LVGFIC and EFC are significantly correlated (R = 0.47), which observation readily follows from involvement of their shared components SV, ESV, and EDV.

**Table 1:** Baseline data for patients, stratified for sex.

	**Women**	**Men**	***P*-value**
**Participants**	18	78	
Age (years)	55 ± 18	51 ± 14	0.34
HR (bpm)	71 ± 14	68 ± 9	0.20
SBP (mmHg)	122 ± 25	132 ± 22	0.08
DBP (mmHg)	72 ± 11	82 ± 13	0.007
BSA (m^2^)	1.65 ± 0.15	1.96 ± 0.15	<0.0001
ESV (mL)	54 ± 24	76 ± 26	0.001
EDV (mL)	108 ± 28	140 ± 31	0.0002
SV (mL)	54 ± 9	64 ± 15	<0.0001
(ESV+EDV)/2 (mL)	81.4 ± 25.7	108.4 ± 27.3	0.0005
EF (%)	51 ± 8	46 ± 9	0.045
EFC (mL)	122 ± 36	160 ± 38	0.0004
LV mass (g)	94 ± 20	124 ± 22	<0.0001
GLS (%)	−10.76 ± 3.47	−9.58 ± 3.32	0.28
LVGFI (%)	32 ± 6	29 ± 7	0.044
LVGFIC (mL)	179.9 ± 37.5	235.6 ± 41.6	<0.0001
MCF (%)	62.3 ± 15.7	55.8 ± 17.7	0.14
MCFC (mL)	105.5 ± 17.3	134.9 ± 20.9	<0.0001
VAC	1.09 ± 0.28	0.92 ± 0.35	0.042
VACC (mL)	77.76 ± 22.13	101.34 ± 23.28	0.0004

Table 2 presents LV volume-related data for the three diagnostic categories under study, along with significance levels calculated for the comparisons among the three groups. The various components (such as SV and average LV volume) that contribute to the formula defining LVGFI (see equation 1) are individually listed, and will later be used for a sensitivity analysis. It appears that there is no single variable or derived index (with the exception of GLS) that can distinguish the three categories, which observation is fully understandable for these dimensionless ratios as they are by definition incomplete. Sex-specific differences may play a role in two ways: a particular variable may not only “by nature” be smaller in one sex (such as LV volume in women), but also in a diagnostic (sub)group because of excessive overrepresentation. For example, in these patients with acute cardiac problems, LV mass is smaller (*P* < 0.0001) in women, but women also constitute the majority (91%) in the takotsubo group.

**Table 2:** Comparison of primary data (ESV, EDV, and LVmass) and their derivatives for the 3 diagnostic groups.

	**AMI**	**Myocarditis**	**Takotsubo**	**P(AMI vs. M)**	**P(AMI vs. TCM)**	**P(M vs. TCM)**
*N*	69	16	11			
Women (%)	9	6	91			
ESV (mL)	77 ± 29	67 ± 16	52 ± 14	0.161	0.026	0.0111
EDV (mL)	137 ± 33	140 ± 28	108 ± 23	0.757	0.003	0.002
(ESV+EDV)/2 (mL)	99 ± 21	107 ± 22	82 ± 17	0.172	0.008	0.002
SV (mL)	60 ± 13	74 ± 18	57 ± 11	0.001	0.185	0.006
EF (%)	45 ± 9	52 ± 7	53 ± 5	0.005	0.005	0.437
EFC (mL)	158 ± 42	155 ± 31	120 ± 27	0.826	0.003	0.002
LV mass (g)	124 ± 23	113 ± 23	89 ± 17	0.082	<0.0001	0.007
LVGFI (%)	27 ± 6	35 ± 9	34 ± 4	0.002	0.001	0.725
LVGFIC (mL)	233.6 ± 45.6	223.4 ± 36.9	174.6 ± 27.8	0.35	<0.0001	0.0006
GLS (%)	−9.06 ± 3.67	−11.00 ± 2.22	−13.10 ± 2.67	0.046	0.0004	0.018

*AMI, acute myocardial infarction; M, myocarditis; TCM, takotsubo cardiomyopathy*.

### Sensitivity Analysis

**Figure 7:**
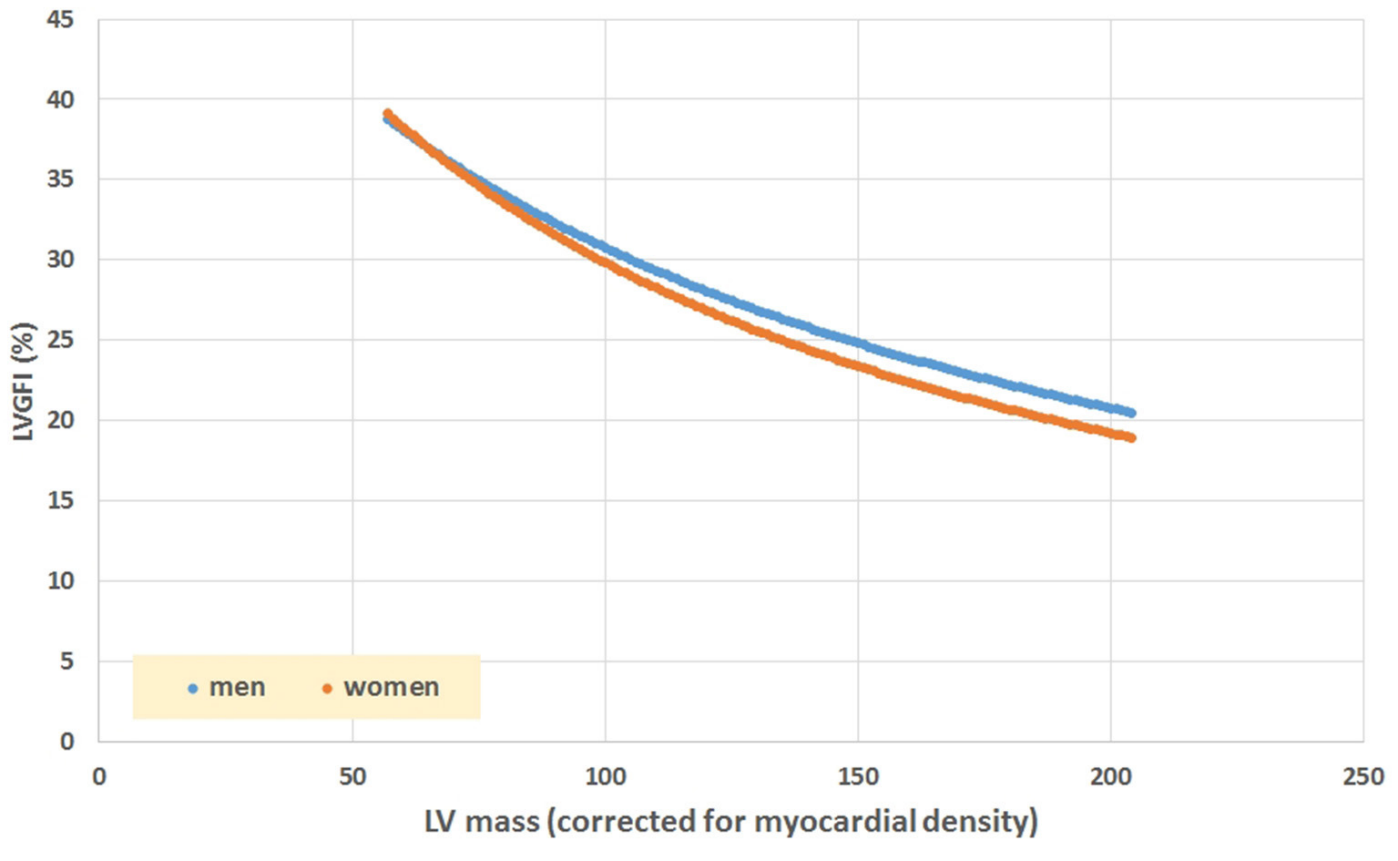
Simulation of LVGFI vs. LV mass (expressed as mL after inclusion of myocardial density), using average values for SV and (EDV+ESV)/2 as obtained in our patients (Table 1), stratified for sex.

**Figure 8:**
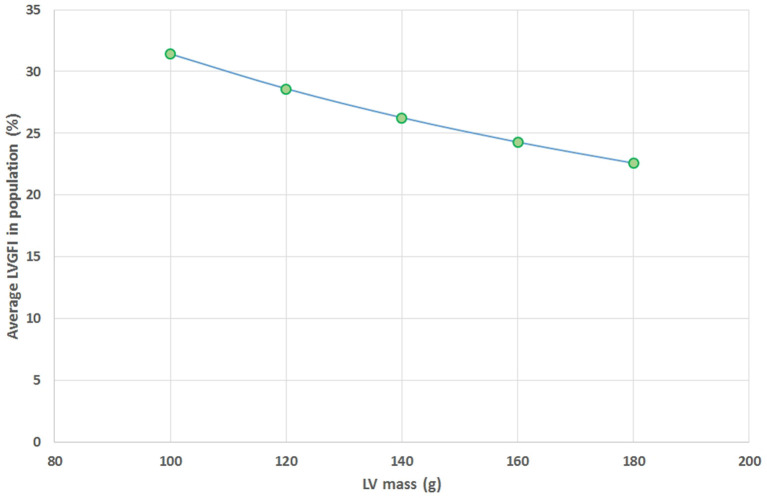
Average values found for LVGFI on the basis of actual patient data for SV, ESV and EDV, but with fixed values for LV mass assumed for all patients (*N* = 96), and set at 100, 120, 140, 160, and 180 grams. This theoretical curve shows a monotonous decline as LV mass increases.

**Figure 9:**
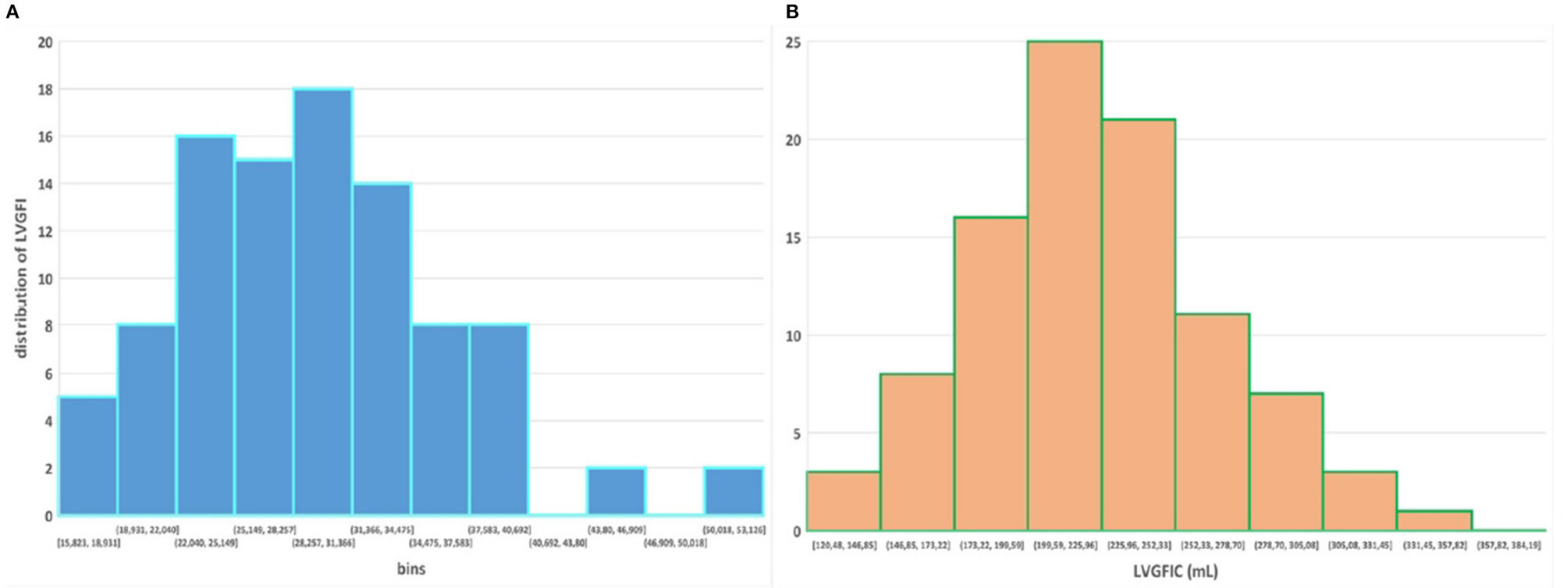
**(A,B)** Histograms for LVGFI and LVGFIC, respectively. Compare with Figure 1 in Nwabuo et al. (2).

**Figure 10:**
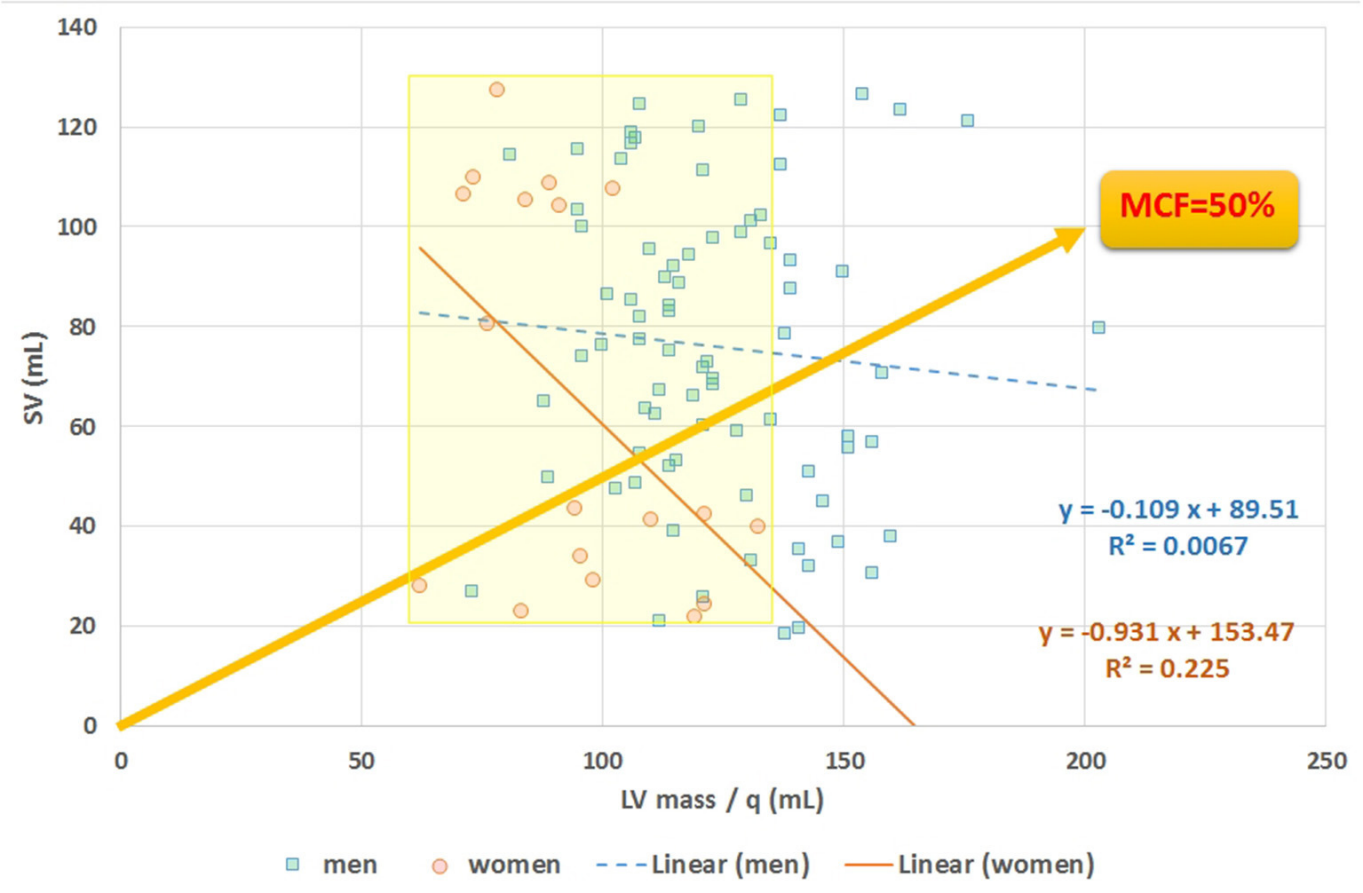
Scatter plot of stroke volume (SV) vs. LVmass. The slope of any line refers to myocardial contraction fraction (MCF), with data points stratified for men and women, as exemplified by the yellow line referring to the case of MCF = 50%. Average MCF in women is not different compared to men, despite their smaller (*P* < 0.0001) LVmass (see yellow marked area).

The mathematical characteristics of LVGFI are theoretically analyzed for the population averaged SV and (ESV+EDV) values in dependence of variation of LVmass for the entire range encountered in this study, i.e., from 62 to 203 grams. Results are shown in Figure 7, indicating that the curves for men and women largely overlap. However, it should be kept in mind that in women the LVmass is significantly smaller (Table 1), meaning that on average the position on the curve is relatively shifted to the left, and implying a higher level of LVGFI as supported by the findings in Table 1. Moreover, Table 1 specifies that mean LVmass in the denominator is slightly larger than the added term (ESV+EDV)/2, implying that the impact of LVmass is generally somewhat more pronounced. Alternatively, we have evaluated the behavior of LVGFI while stepwise varying the LVmass variable, and applying the actual patient-based values for SV, ESV and EDV. The results indicate that the impact of LVmass varies, with the highest contribution to LVGFI in the lower LV mass range (Figure 8). Histograms for LVGFI(C) are shown in Figures 9A,B. The more simple index MCF is further explored in Figure 10, and illustrates the ratio between SV and LVmass/q without considering the (ESV+EDV)/2 term, in contrast to the definition of LVGFI.

## Discussion

We have dissected the definition formula of LVGFI by separately considering its components. First inspection reveals that the definition includes redundant elements, as SV is the difference between EDV and ESV, which pair returns again as their sum (ESV+EDV)/2. Part of this duplication is then mixed by considering their ratio, which construct introduces another problem, namely the complete removal of physical dimensions. The route followed here would be equivalent to dividing pulse pressure by mean pressure in case of hemodynamics, where the latter variable in fact is known to correspond with the companion to pulse pressure itself (9). Thus, a particular metric is divided by its associated companion in order to obtain a single number, rather than the two components being analyzed in unison. Interestingly, EF (which by definition is proportional to SV = [EDV–ESV]), is also inversely tied to the sum of ESV and EDV (Figure 6). The analysis of our patient data confirms the theoretical weakness of LVGFI by demonstrating the impact of the associated LVGFIC. Also, EDV correlates well (R = 0.98) with mean LV volume, meaning that LVGFI can be redefined as SV/(LVmass/q+EDV), which resembles the definition of EF, where the denominator is modulated by LVmass/q (in our study 118.2 ± 24.7 with range 62–203 g). Indeed, a potentially useful contributor in the definition formula for LVGFI concerns the LVmass term, although it remains unclear why this element is introduced as an additive component in the denominator. Anyhow, we performed a sensitivity analysis, and found that curves for both sexes overlap, but actual working points for women are shifted to the left (Figure 7).

Considering the fact that the (ESV+EDV) term in LVGFI is associated with EF (Figure 6), it is clear why Reinstadler et al. (10) found that in STEMI patients c-statistics revealed that LVGFI does not provide incremental prognostic information over EF (*P* = 0.38), although being a predictor of major adverse cardiac events (MACE). However, also in a group of STEMI patients treated with primary percutaneous coronary intervention Eitel et al. (11) found that LVGFI was associated with infarct size and did show an incremental prognostic value in addition to EF for prediction of all-cause mortality, rather than for MACE.

Obviously, a single component may dominate the ratio depending on the type of patients studied, e.g., LVmass was higher in HF patients compared to those with no events in the study by Nwabuo et al. (2). Likely for that reason it was found that LVGFI is a strong, independent predictor of incident HF and cardiovascular disease that provides incremental prognostic value compared with EF.

Sex-specific differences also deserve attention. In equation 1 the smaller SV generally found in women (*P* < 0.0001 in our Table 1) largely cancels out against the concomitant lower values for mean LV volume (*P* = 0.0005 in our Table 1). In fact, SV also has a companion, which happens to equal the one found for EFC (3). In addition, the term (ESV+EDV) has a quadratic mean companion, as does the denominator (D), yielding DC = √ [(LVmass/q)^2^ + ((ESV+EDV)/2)^2^], as e.g., D = 200 may result from LVmass/q is 100 and (ESV+EDV)/2 is 100, or the combination 80 and 120, etc. For those combinations the DC would be 141.4 and 128.1 mL, respectively.

It can be concluded that LVGFI does not carry any “magic power” beyond the traditional metric EF. The impact of the term “SV” in the formula is already partly covered by the combination of ESV and EDV. The only extra element introduced in the definition formula compared to the familiar EF concerns LVmass. Of course, LVmass is a relevant piece of information. However, physiology does not refer to the art of mixing various variables into a single mathematical construct, and certainly not in case of a dimensionless composition. Numerous variations on this theme can be developed.

A recent example concerns a modification that accounts for unique loading conditions in patients with repaired tetralogy of Fallot by incorporating “effective SV” (eSV) in order to derive RV effective GFI (eGFI) (12). This extra input makes sense, as eSV must be *measured*, and cannot not be calculated from ESV and EDV. However, mixing eSV in a ratio-based metric again urges the necessity of invoking the corresponding companion for this particular situation.

Specific applications have been reported, e.g., Huang et al. (13) found that LVGFI is a clinically useful parameter with excellent ability in determining myocardial function and differentiating cardiac amyloidosis from hypertrophic cardiomyopathy, and Mele et al. (14) used speckle-tracking echocardiography to predict LV remodeling after acute ST-segment elevation myocardial infarction.

MCF = SV.q/LVmass, introduced by King et al. (5), is also an index that combines global systolic performance with anatomic information. Compared to the expression for LVGFI the LV volume term (ESV+EDV)/2 has here been eliminated from the denominator. Abdalla et al. (15) employed this reduced measure in an CMRI study that included 5,000 patients and found that this metric can be used to predict incident events such as myocardial infarction, resuscitated cardiac arrest, stroke, coronary heart disease related death, and stroke death. In our study MCF is associated with both LVGFIC (R = 0.65) and with EF (R = 0.58). Furthermore, for our data MCF is unrelated (with R < 0.03 for men and women) to the term (ESV+EDV)/2.

Most studies on EF report on population-based volumetric data, while only a few publications present the dynamic behavior of volume regulation for a single heart. However, sequential data collection in individuals is common practice following heart transplantation, and a recent case report documents the superiority of ESV above ratios (such as EF or VAC) when evaluating the long term course of these patients (16). Interestingly, that study documents that one of the two components involved in the EF ratio (namely ESV) is able to perform better than the composite ratio-based construct.

The bottom line is that the creation of any novel ventricular function index that includes more than what is embodied by EF itself (i.e., merely a ratio of ESV and EDV), will one way or the other demonstrate (some) incremental value, just because EF is by definition incomplete (see Appendix). The challenge then is to demonstrate that the novel index has something to offer beyond the *combination* of EF and EFC, or being superior to the *combination* of ESV and EDV. In case LVmass is selected as a precious addition, it may in the future be relevant to consider ESV, EDV and LVmass in a 3-dimensional framework.

## Data Availability Statement

The raw data supporting the conclusions of this article will be made available by the authors, without undue reservation.

## Ethics Statement

The studies involving human participants were reviewed and approved by the local Ethics Committee of Reñaca Clinic. The patients/participants provided their written informed consent to participate in this study.

## Author Contributions

RD-N and PK contributed to conception and design of the study and organized the database. PK performed the statistical analysis and wrote the first draft of the manuscript. RD-N wrote sections of the manuscript. All authors contributed to manuscript revision, read, and approved the submitted version.

## Conflict of Interest

The authors declare that the research was conducted in the absence of any commercial or financial relationships that could be construed as a potential conflict of interest.

## Publisher's Note

All claims expressed in this article are solely those of the authors and do not necessarily represent those of their affiliated organizations, or those of the publisher, the editors and the reviewers. Any product that may be evaluated in this article, or claim that may be made by its manufacturer, is not guaranteed or endorsed by the publisher.
