# Supplementary material for: Left Ventricular Global Function Index and the Impact of its Companion Metric
Source: Front Cardiovasc Med. 2021 Aug 30;8:695883. doi: 10.3389/fcvm.2021.695883 (PMC8435684; doi:10.3389/fcvm.2021.695883)
Supplement: Supplementary file 1 [file Data_Sheet_1.docx]

# APPENDIX

**Left Ventricular Global Function Index and the Impact of its Companion Metric** (doi: 10.3389/fcvm.2021.695883)

**Rienzi A. Diaz-Navarro and Peter L.M. Kerkhof**

This section explores the impact of various components of the left ventricular (LV) global function index (LVGFI) by selectively eliminating terms or introducing substitutes. The LVGFI is defined as a composite of various volumetric variables (including the sum of ESV and EDV) and LVmass:

LVGFI = SV / {LVmass/q + 0.5*(ESV+EDV)} eq (A1)

As these variables are components of a ratio, some simplifications can be introduced. First, we can altogether leave out the average volume term (ESV+EDV)/2, resulting in another popular (dimensionless) index, namely MCF:

MCF = SV*q / LVmass eq (A2)

Compared to eq (A1) this reduced expression misses the term that refers to EF (see eq. A4), and actually indicates the amount of blood (mL) ejected per beat relative to the LV myocardial tissue volume (mL) involved. Importantly, SV is a difference that does not disclose under which operating conditions (i.e. referring to EDV and ESV) this particular SV is generated. In chronic pathology cases it is likely that LVmass increases with ventricular enlargement, thus potentially reflecting LV size.

Furthermore, LVGFI can be rewritten by substitution of the definition formula for EF, yielding:

LVGFI = EF / {LVmass/q*EDV – 1 + EF/2} eq (A3)

where EF is expressed as a fraction (between 0 and 1). Here, LVGFI explicitly depends on EF, EDV and LVmass, besides the constant q.

Yet another route, not generally recognized, can be followed by relating EF to the average value of LV volume, taken as (maximum + minimum)/2. This simple mathematical choice may clarify several clinical observations. Indeed, it is known that (ESV+EDV)/2 linearly correlates with EF, thus

(ESV+EDV)/2=a+bEF eq (A4)

yielding

LVGFI = 100*SV / [0.95*LVM+a+bEF] eq (A5)

Substituting SV=EDV*EF/100 and ESV=p+qEDV gives:

LVGFI = (ESV-p) *EF/q*[0.95*LVM+a+bEF] eq (A6)

Furthermore EF = 100*{1-c_1_ ESV/ (ESV-c_2_)}*,* where c_1_ and c_2_ depend on a, b, average EDV, and the correlation coefficient found for ESV vs EDV (S1).

Thus, LVGFI = f (ESV, LVM, a, b, p, q, c1, c2)

If LVM/q is assumed fixed around 100 and left out, then we find LVGFI = SV/EF ~ EDV, in other words actual LVGFI equals “EDV modulated by the impact of LVM/q” (where LVM/q by itself may depend on EDV).

The companion (C) of LVGFI is defined as (S2):

LVGFIC = √ {(100*SV)^2^+(0.95*LVM+0.5*(ESV+EDV))^2^} eq (A7)

On the other hand:

EF = 1 - {(ESP / Emax) + Vo} / {(log EDP – log Po) / k} eq (A8)

with the volume intercept (Vo) of the end-systolic elastance curve (S3). Furthermore we estimated ventriculo-arterial coupling VAC as

VAC = SV/ESV eq (A9)

while assuming that the Vo vanishes.

Figure S1 summarizes the primary variables and derived metrics.

**Figure S1.** Schematic diagram illustrating the connections between basic variables (LVmass, ESV and EDV) and derived metrics, including SV, EF, LVGFI and MCF. All derived metrics carry an associated companion.

## Supplemental References:

S1. Kerkhof PLM, van de Ven PM, Yoo B, Peace RA, Heyndrickx GR, Handly N. Ejection fraction as related to basic components in the left and right ventricular volume domains. *Int J Cardiol*. 2018 Mar 15;255:105-110. doi: 10.1016/j.ijcard.2017.09.019.

S2. Kerkhof PLM, Peace RA, Handly N. Ratiology and a Complementary Class of Metrics for Cardiovascular Investigations. *Physiology (Bethesda)*. 2019 Jul 1;34(4):250-263. doi: 10.1152/physiol.00056.2018.

S3. Kerkhof PLM, Yoo BW, Merillon JP, Peace RA, Handly N. Monte Carlo method applied to the evaluation of the relationship between ejection fraction and its constituent components. *Annu Int Conf IEEE Eng Med Biol Soc*. 2017 Jul;2017:1295-1298. doi: 10.1109/EMBC.2017.8037069.
